# Advanced Neuroimaging Preceding Intravenous Thrombolysis in Acute Ischemic Stroke Patients Is Safe and Effective

**DOI:** 10.3390/jcm10132819

**Published:** 2021-06-26

**Authors:** Klearchos Psychogios, Apostolos Safouris, Odysseas Kargiotis, Georgios Magoufis, Athina Andrikopoulou, Ermioni Papageorgiou, Maria Chondrogianni, Georgios Papadimitropoulos, Eftihia Polyzogopoulou, Stavros Spiliopoulos, Elias Brountzos, Elefterios Stamboulis, Sotirios Giannopoulos, Georgios Tsivgoulis

**Affiliations:** 1 Acute Stroke Unit, Metropolitan Hospital, Ethnarhou Makariou 9, 18547 Piraeus, Greece; safouris@yahoo.com (A.S.); kargiody@gmail.com (O.K.); magoufisgeorge1@icloud.com (G.M.); andriathi@yahoo.gr (A.A.); erminapapageorgiou@yahoo.gr (E.P.); mariachondrogianni@hotmail.gr (M.C.); ge.papadim@gmail.com (G.P.); lstam@med.uoa.gr (E.S.); 2 Second Department of Neurology, National & Kapodistrian University of Athens, School of Medicine, “Attikon” University Hospital, 12462 Athens, Greece; sgiannop@uoi.gr (S.G.); tsivgoulisgiorg@yahoo.gr (G.T.); 3 Emergency Medicine Clinic, National & Kapodistrian University of Athens, School of Medicine, “Attikon” University Hospital, 12462 Athens, Greece; effiepol@med.uoa.gr; 4 Second Department of Radiology, Interventional Radiology Unit, “ATTIKON” University General Hospital, 12462 Athens, Greece; stavspiliop@med.uoa.gr (S.S.); eliasbrountzos@gmail.com (E.B.)

**Keywords:** acute stroke, intravenous thrombolysis, perfusion imaging, CT perfusion, MR perfusion, RAPID

## Abstract

Advanced neuroimaging is one of the most important means that we have in the attempt to overcome time constraints and expand the use of intravenous thrombolysis (IVT). We assessed whether, and how, the prior use of advanced neuroimaging (AN), and more specifically CT/MR perfusion post-processed with RAPID software, regardless of time from symptoms onset, affected the outcomes of acute ischemic stroke (AIS) patients who received IVT. Methods. We retrospectively evaluated consecutive AIS patients who received intravenous thrombolysis monotherapy (without endovascular reperfusion) during a six-year period. The study population was divided into two groups according to the neuroimaging protocol used prior to IVT administration in AIS patients (AN+ vs. AN−). Safety outcomes included any intracranial hemorrhage (ICH) and 3-month mortality. Effectiveness outcomes included door-to-needle time, neurological status (NIHSS-score) on discharge, and functional status at three months assessed by the modified Rankin Scale (mRS). Results. The rate of IVT monotherapy increased from ten patients per year (*n* = 29) in the AN− to fifteen patients per year (*n* = 47) in the AN+ group. Although the onset-to-treatment time was longer in the AN+ cohort, the two groups did not differ in door-to-needle time, discharge NIHSS-score, symptomatic ICH, any ICH, 3-month favorable functional outcome (mRS-scores of 0–1), 3-month functional independence (mRS-scores of 0–2), distribution of 3-month mRS-scores, or 3-month mortality. Conclusion. Our pilot observational study showed that the incorporation of advanced neuroimaging in the acute stroke chain pathway in AIS patients increases the yield of IVT administration without affecting the effectiveness and safety of the treatment.

## 1. Introduction

Intravenous thrombolysis (IVT) with alteplase in acute ischemic stroke (AIS) administered within the first 4.5 hours following symptom onset remains the mainstay of acute reperfusion therapies [1,2,3]. Despite tissue plasminogen activator (tPA) effectiveness, only a small number of AIS patients worldwide benefit from IVT [4,5]. Short therapeutic time window, strict inclusion and exclusion criteria of the pivotal randomized controlled clinical trials (RCTs), as well as health care system disparities, such as public awareness on how to act in case of stroke symptoms, organization of emergency medical services, and the paucity of organized stroke centers in rural areas [6], have been significant barriers to overcome. Nevertheless, off-label use of IVT [7,8] is increasingly incorporated in the everyday clinical practice of many stroke practitioners.

Advanced neuroimaging may help us overcome time constraints and expand the implementation of acute reperfusion therapies [9]. CT and MR perfusion with automated post-processing software (RAPID, iSchemaView, Menlo Park, CA, USA) have proven effective in recent RCTs, for both mechanical thrombectomy candidates in the late time window (6–24 h) [10,11] and for IVT (4.5–9 h and wake-up patients) [12,13,14]. Advanced neuroimaging provides a ”brain physiology snapshot in time” that can guide decisions for recanalization therapies in clinical practice [15]. Numerous stroke centers and stroke units worldwide have incorporated the use of CT and MR perfusion in their acute therapeutic pathways.

In view of the former considerations, we assessed the differences in the use of IVT monotherapy and the outcomes of the AIS patients with or without the use of advanced neuroimaging.

## 2. Materials and Methods

We retrospectively evaluated consecutive AIS patients who received IVT admitted to our European Stroke Organization certified stroke unit. We also participate in the SITS (Safe Implementation of Thrombolysis in Stroke) and RES-Q (Registry of Stroke Care Quality) international registries [16,17]. Patients were included if they fulfilled the following criteria: (1) aged over 18 years old; (2) clinically diagnosed with AIS with a measurable neurologic deficit on the National Institute of Health Stroke Scale (NIHSS) presenting within the 4.5 h window from symptom onset; (3) AIS patients were considered eligible for the extended time window of 4.5–9 h if they presented after 4.5 h and sooner than 9 h from last-seen-well (late window patients), according to the clinical and neuroimaging inclusion criteria of the EXTEND trial [10]; (4) AIS patients who woke up with symptoms of stroke («wake-up stroke») were treated according to the WAKE-UP trial [18] protocol; and (5) AIS patients treated with IVT monotherapy. All patients with large vessel occlusion (LVO) who underwent mechanical thrombectomy were excluded. Transient ischemic attacks and stroke mimics were excluded from the current study based on clinical and neuroimaging criteria.

The study population was divided into two different groups according to the neuroimaging protocol used on admission and prior to IVT administration in AIS patients (with prior Advanced Neuroimaging (AN+) vs. without prior advanced neuroimaging (AN−)). Of note, the neuroimaging protocol was modified in our center on December 2017 after the introduction of perfusion imaging (with RAPID software) and on August 2018 after the publication of the WAKE-UP trial. Patients in the first study group (AN−) underwent baseline emergent neurovascular imaging using either non-contrast-enhanced computed tomography (NCCT), with or without CT angiography (CTA), or magnetic resonance imaging (MRI) with magnetic resonance angiography based on the treating physician’s decision. Patients in the second study group (AN+) underwent NCCT/CTA/computed tomography perfusion (CTA/CTP) or magnetic resonance angiography/magnetic resonance perfusion (MRA/MRP) unless they presented certain contraindications (e.g., renal insufficiency, severe allergic reactions to iodinised agents, etc.). CT perfusion was performed using two continuous 2.5 cm slabs, starting at the level of the circle of Willis for most patients, lower for those presenting with symptoms suggesting posterior fossa ischemia, and higher for those presenting with symptoms suggestive of cortical ischemia. Ischemic core (rCBF < 30%), critically hypoperfused ischemic region (Tmax > 6 s), and mismatch volume corresponding to ischemic penumbra, were estimated by using RAPID as previously described [19]. The hyperdense vessel sign (HVS), a highly specific marker of arterial obstruction [20], was identified on non-contrast CT if the lumen of any, non-calcified, intracranial artery appeared denser than adjacent or equivalent contralateral arteries. Clot length was quantified based on CT angiography by using standard methodology [21]. The LVO was defined as the occlusion of the internal carotid artery (ICA), basilar artery (BA), and the first segment of the Middle Cerebral Artery (MCA-M1). CT/MR findings were interpreted and extracted independently by experienced neurologists or neuroradiologists that were blinded to clinical outcomes.

The following parameters were recorded for all included patients: (1) demographic characteristics; (2) history of vascular risk factors (diabetes mellitus, hypertension, current smoking, hypercholesterolemia, coronary artery disease, peripheral artery disease, congestive heart failure, and valvular disease) as previously described [22]; (3) prior history of stroke or Transient Ischemic Attack (TIA); (4) laboratory test values on admission (total platelet count, glucose, and low-density lipoprotein (LDL) levels); and (5) admission systolic and diastolic blood pressures, measured using automated blood pressure cuffs. Stroke severity was assessed with the NIHSS (National Institute of Health Stroke Scale) score at admission, 2 h and 24 h post IVT, and at discharge. Safety outcomes included prevalence of symptomatic intracranial hemorrhage (sICH), prevalence of any intracranial hemorrhage in the 24-h post thrombolysis neuroimaging studies, and 3-month mortality. sICH was defined using standard SITS registry definitions (local or remote parenchymatous hemorrhage type 2 combined with an NIHSS-score increase of >4 points or leading to death\22–36 h) [14]. Any intracranial hemorrhage was recorded according to the ECASS criteria [23]. Effectiveness outcomes included door-to-needle time, neurological improvement at 24 h and on discharge, and functional status at discharge and at 3 months by using the modified Rankin Scale (mRS). Functional independence (FI) and favorable functional outcome (FFO) were defined as an mRS-score of 0–2 or an mRS-score of 0–1 at 3 months, respectively. Stroke severity and functional outcome (mRS) at discharge and at 3 months were assessed by certified vascular neurologists as previously described [24].

All follow-up evaluations occurred at 90 ± 10 days from symptom onset at the Stroke Outpatient Clinic of our institution as previously described [25]. The evaluation of the mRS-score was performed by certified vascular neurologists who were unaware of the neuroimaging protocol that was implemented at baseline.

### Statistical Analysis

All binary variables were presented as percentages, while continuous variables were presented with their corresponding mean values and standard deviations (SDs), in cases of normal distributions, or as medians with interquartile ranges (IQRs) in cases of skewed distributions. Statistical comparisons between the two groups were performed using the unpaired t test, Mann–Whitney U-test, *χ*^2^ test, and Fisher exact test, as appropriate. The distribution of the 3-month mRS scores between patients treated before and after RAPID implementation was compared using the Cochran–Mantel–Haenszel test and the univariable/multivariable ordinal logistic regression (shift analysis).

All efficacy and safety outcomes of interest were further assessed in univariable and multivariable binary logistic regression models adjusting for the a priori defined confounders of the age and baseline NIHSS-score. The final variables that were independently associated in the multivariable logistic and the ordinal regression analyses with the outcome of interest, were selected using an alpha value of 0.05 and adjusted associations were provided as odds ratios (ORs) or common odds ratios (cORs), with their corresponding 95% confidence intervals (95% CI).

All statistical analyses were conducted with the Stata Statistical Software Release 13 (StataCorp LP, College Station, TX, USA).

## 3. Results

**Figure 1:**
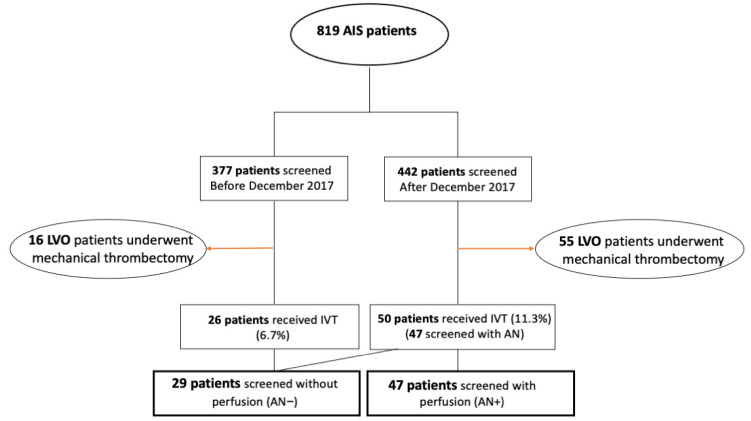
Flowchart of the study population. Acute Ischemic Stroke, AIS; Large vessel occlusion, LVO; intravenous thrombolysis, IVT; Advanced Neuroimaging, AN.

A total of eight hundred and nineteen patients were screened in the setting of an acute stroke code between February 2015 and January 2021. The complete flowchart of our study is shown in Figure 1. Three hundred and seventy-seven patients were screened before December 2017 (AN implementation) and twenty-six received IVT, whereas four hundred and forty-two were screened after December 2017 and fifty patients among them received IVT (three of them were not screened with prior AN due to contraindications). Our final cohort was comprised of 76 AIS patients who received IVT throughout the entire study period. All patients who received endovascular reperfusion therapy with mechanical thrombectomy were excluded from our analysis (*n* = 71). Twenty-nine patients received IVT without prior advanced neuroimaging (AN−) and forty-seven patients with the use of advanced neuroimaging (AN+). The rate of IVT monotherapy increased from ten patients per year in the AN− to fifteen patients per year in the AN+ group. Baseline characteristics of the two treatment groups are summarized in Table 1. Patients in the AN+ group were significantly (*p* = 0.003) older than patients in the AN− group (mean age 73 years vs. 63 years, respectively). Median admission NIHSS-scores were 4 points (IQR: 2–7) in the AN− group and 5 points (IQR: 4–9) in the AN+ group, a difference that was also significant (*p* = 0.047). The prevalence of large vessel occlusions was 17.2% in the AN− group and 19.1% in the AN+ group (*p* = 0.835). The location of stroke in posterior circulation was more frequent in the AN− group (34.5%) than in the second study group (19.1%). The median elapsed time between symptom onset (or last-seen-well) to initiation of IVT was significantly longer in the second group (198 min (IQR: 151–240)) in AN+ vs. 121 min ((IQR: 130–220) in AN−; *p* < 0.001), whereas the door-to-needle time was almost identical between the two groups (median 44 min (IQR: 36–60)) in AN− vs. 45 min ((IQR: 30–61) in AN+; *p* = 0.956). The rate of patients treated according to the EXTEND trial or WAKE-UP protocol was significantly higher in the second study group (23.4% vs. 3.4%; *p* = 0.020). All patients were treated with alteplase, except for four patients in the AN+ with large vessel occlusions who were treated with tenecteplase.

The neuroimaging characteristics are summarized in Table 1 and Table 2. The median thrombus length tended to be higher in the AN+ group (12 vs. 9 mm, *p* = 0.053). The median ASPECTS score and the presence of a hyperdense vessel sign were similar across the study groups. MR imaging was performed in 6.9% of AN− and 10.3% of AN+ patients (*p* = 0.584). In patients who underwent perfusion imaging, the mean ischemic core volume was calculated at 2.1 ± 1.2 mL and the mean volume of critical hypoperfusion was 16.3 ± 4.0 mL (Table 2).

Table 3 summarizes the effectiveness and the safety outcomes in the two patient groups. There was only one missing 3-month follow-up evaluation in each treatment group. Neurological status assessed by NIHSS at 2 h, 24 h, and at hospital discharge was similar between the two groups. The rates of sICH (3.4% vs. 0%; *p* = 0.2) and any intracranial hemorrhage (6.9% vs. 10.6%; *p* = 0.584) were similar between the two groups. The rates of 3-month favorable functional outcome (75% vs. 78.3%; *p* = 0.746), 3-month functional independence (82.1% vs. 89.1%; *p* = 0.394), and 3-month mortality (0% vs. 4.3%; *p* = 0.263) did not differ between the two groups either. A secondary analysis restricted to the patients in the early time window shows similar results (Supplemental Table S1).

The distribution of 3-month mRS-scores was similar between the two groups (*p* for Cochran–Mantel–Haenszel test: 0.466). Table 4 shows the univariable and multivariable associations of the neuroimaging protocol with safety and efficacy outcomes in multivariable logistic regression models adjusting for the age and admission NIHSS-score. There was no association between the advanced neuroimaging protocol and any ICH (crude OR 1.60, 95% CI: 0.29–8.88; *p* = 0.586), functional independence at three months (crude OR 1.78, 95% CI: 0.47–6.8; *p* = 0.398), and favorable functional outcome at three months (crude OR 1.20, 95% CI: 0.40–3.63; *p* = 0.747). In adjusted analysis AN was associated with better functional independence at 3 months (adjusted OR 12.89, 95% CI: 1.47–113; *p* = 0.021).

## 4. Discussion

Our pilot observational single-center study showed that the shift in our clinical practice, with the incorporation of advanced neuroimaging in AIS patients, increases the yield of IVT administration by approximately 50% without major effectiveness and safety repercussions. On the contrary, all comparisons showed that it is equally safe, and even in a population with more negative prognostic factors (higher admission NIHSS-score, older age, longer thrombus), we documented a trend towards better functional outcomes without any delays in door-to-needle time. Better outcomes in patients with prior AN possibly reflect the comparison between different study periods and the accumulating experience of the stroke team through the years. It might also encompass the more favorable prognosis of patients treated in the extended time window, already proven by large clinical trials [12]. However, this result should be treated with caution given the large confidence intervals due to our small study sample and the fact that it was not demonstrated in the crude analysis as well.

**Figure 2:**
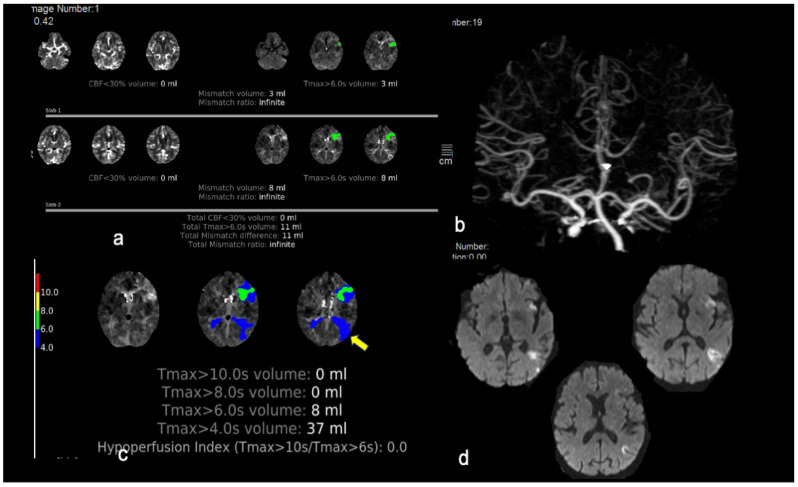
This is an illustrative case of a patient fulfilling both neuroimaging and clinical EXTEND eligibility criteria who was treated successfully with intravenous thrombolysis in the extended time window. An 80-year-old woman was transferred from an island to the emergency department 5 h after an acute onset of expressive aphasia, mild right facial paresis, and mild right upper arm paresis (ΝΙHSS score 9 points). (**a**) Her CT-perfusion mismatch map post-processed with RAPID software demonstrated a hypoperfused region of 11 mL in the Broca’s area (shown in green) and no area of reduced cerebral blood flow, resulting in a 11 mL mismatch difference (infinite mismatch ratio). (**b**,**c**) CT angiogram revealed no large vessel occlusion. The patient fulfilled all EXTEND eligibility criteria; IVT with alteplase started 5 h and 45 min after symptom onset with partial resolution of symptoms at the end of tPA infusion (NIHSS-score of 6 points). (**d**) Repeat MRI at 24 h demonstrated a small insular infarct and another acute infarct in the left temporoparietal region which was captured in the Tmax maps of initial perfusion imaging as Tmax > 4 s prolongation (**c**/arrow). The patient’s mRS-score at three months was 0.

Almost 25% of patients in the advanced neuroimaging group were treated based on neuroimaging criteria (either extended time window 4.5–9 h or wake-up strokes, see Figure 2) and this further substantiates our previous observations [20]. Considering that the extra time needed to perform the CT perfusion and to acquire the RAPID templates is at least ten min, it is striking that the median door-to-needle time was only one min longer in the advanced neuroimaging group compared to the median door-to-needle time in the standard neuroimaging group. This observation reflects the interplay of many other important key factors: the acquired experience of the personnel who are involved in the acute stroke chain, the increased use of perfusion imaging particularly in “borderline” cases (e.g., stroke mimics) [26] that otherwise would necessitate two different imaging modalities (CT and MRI), and the fact that the clinical decision in most cases was made immediately after the non-contrast CT and IVT could be initiated in the radiology department before completion of the perfusion imaging.

The present study investigated the effect of advanced neuroimaging on IVT monotherapy. Patients who received endovascular reperfusion therapy were excluded from our analyses. Consequently, our cohort included predominantly mild to moderate severity strokes with a small ischemic core and penumbra volumes or patients with LVO who responded to IVT with successful reperfusion and did not need further endovascular treatment. This probably induces a selection bias by excluding AIS patients with a more “unfavorable prognosis”. Previous studies [27,28] that served as pilot studies for the major MT RCTs, have underscored the feasibility of this physiologic imaging approach in cases with LVO-attributed ischemic stroke. Major RCTs that also used the same approach in the early time window [29,30] showed even greater treatment effects, substantially enhancing the use of this approach in clinical practice.

The use of perfusion imaging in AIS patients who present in the first 4.5 h after symptoms onset is still controversial. In our cohort, patients who did not present with a favorable profile (based on neuroimaging criteria) in the early time window, were still offered tPA according to current recommendations. The majority of these patients (n = 19) had no ischemic core or had only hypoperfusion that did not meet the Tmax > 6 s typical criteria of the penumbra. Some of these patients (4/19, 21%) had a “benign oligemia” profile with Tmax prolongation > 4 s, but with either ongoing clinical symptoms or symptoms in partial resolution. This could be due to technical issues (lesion outside the selected slabs when CT perfusion was used), lacunar infarcts [31], spontaneous recanalization before imaging, or small lesions in the posterior circulation [32] where CT perfusion has lower sensitivity. However, it may also imply that among the “benign oligemia” regions, there might exist grey zones close to the Tmax 6 s threshold delay that correspond more to critical hypoperfused areas, and which, if left untreated, may lead to permanent neurological deficits. Indeed, the DEFUSE study [33] showed that among patients who did not experience early reperfusion, Tmax > 4 s threshold was more accurate in predicting final infarct volume. Even though Tmax > 6 s has been proven to be the best perfusion measurement marker in predicting clinical outcome [34,35] after successful recanalization, infarct growth is perhaps a more complex process influenced by many clinical and pathological factors.

Based on current knowledge, perfusion imaging may not be critical for therapeutic decisions in the early time window by excluding patients with large ischemic core or those with no or minimal perfusion deficit. For instance, the “too good to treat” pattern [36] of small distal perfusion lesions with no vessel occlusion, needs to be studied in larger populations and with more potent thrombolytic agents, including tenecteplase. Even though time since last-seen-well is a poor proxy for perfusion status, we are far from changing the paradigm of IVT administration and endovascular treatment in the early time window from time-based to imaging-based. Nevertheless, in the era of precision medicine and shared decision-making [37], perfusion imaging may still provide additional support to the clinician: for instance, to communicate the decisions with the patient and the patient proxies, strengthen the diagnostic confidence by excluding stroke mimics, accelerate the processes in fast-progressors, and possibly, predict prognosis.

Certain limitations of the present pilot study need to be acknowledged including the single-center retrospective design and analysis of a prospectively maintained patient database, the relatively small sample size, the lack of randomization, and blinding in the evaluation of clinical outcomes. In addition, a major limitation is the heterogeneity induced by the comparison of data from different time periods where practices and experiences of the involved personnel are changing and protocols are reviewed and updated periodically.

## 5. Conclusions

In conclusion, the implementation of advanced neuroimaging in unselected AIS patients receiving reperfusion monotherapy with IVT, results in an increase of tPA administration rates without delaying door-to-needle time and without raising safety or effectiveness concerns.

## Figures and Tables

**Table 1:** Baseline characteristics in patients treated before and after the implementation of advanced neuroimaging.

Baseline Characteristics	AN− (*n* = 29)	AN+ (*n* = 47)	*p*-Value
Age, years (mean, SD)	63 ± 16	73 ± 13	0.003
Weight, kg (mean, SD)	82 ± 18	80 ± 21	0.631
Smoking (%)	27.6%	25.5%	0.850
Hypertension (%)	72.4%	57.4%	0.189
Diabetes (%)	31.0%	17.0%	0.154
Hypercholesterolemia (%)	27.6%	42.6%	0.189
Prior stroke (%)	3.4%	4.3%	0.861
Prior TIA (%)	0.0%	6.4%	0.165
Congestive heart failure (%)	3.0%	0.0%	0.200
Valvular disease (%)	6.9%	0.0%	0.068
Coronary artery disease (%)	10.3%	4.3%	0.298
Peripheral Arterial Disease (%)	3.4%	6.4%	0.578
Extended window 4.5–9 h (%)	0.0%	14.9%	0.029
Wake up stroke (%)	3.4%	8.5%	0.387
Extended window or wake up	3.4%	23.4%	0.020
NIHSS-score on admission, points (median, IQR)	4 (2–7)	5 (4–9)	0.047
Systolic BP on admission, mmHg (mean ± SD)	152 ± 34	153 ± 21	0.837
Diastolic BP on admission, mmHg (mean ± SD)	80 ± 15	82 ± 14	0.549
Platelet count on admission, ×10^9^/L (mean ± SD)	267 ± 152	228 ± 83	0.477
LDL on admission, mg/dL (mean ± SD)	137.5 ± 49	129.5 ± 34	0.554
Glucose on admission, mg/dL (mean ± SD)	129 ± 39	129 ± 39	0.174
Onset-to-imaging time, min (median, IQR)	105 (87.5–161)	160 (120–202.5)	0.011
Door-to-needle time, min (median, IQR)	43.5 (36–60)	45 (30–61)	0.956
Onset-to-treatment time (median, IQR)	121 (110–153)	197.5 (151–240)	<0.001
ASPECTS (median, IQR)	10 (9–10)	10 (9–10)	0.278
Duration of Hospitalization (median, IQR)	10 (8–18)	9.5 (5–16.5)	0.725
Location of stroke in the left hemisphere (%)	43.2%	56.8%	0.564
Location of stroke in posterior circulation (%)	34.5%	19.1%	0.199
Hyperdense vessel sign in CT (%)	3.6%	4.3%	0.870
MR imaging (%)	6.9%	10.6%	0.584
Thrombus length, mm (median, IQR)	8.5 (5.75–14)	12 (9–20)	0.053
Large Vessel occlusion (%)	17.2%	19.1%	0.835
Medium Vessel Occlusion (%)	44.8%	40.4%	0.706

Blood pressure, BP; National Institute of Health Stroke Scale, NIHSS; interquartile range, IQR; Alberta Stroke Program Early CT score, ASPECTS; standard deviation, SD.

**Table 2:** Neuroimaging characteristics of patients treated after the implementation of perfusion imaging.

Mean ischemic core volume (rCBF < 30%) (mean ± SD) (mL)	2.1 ± 1.2
Mean volume of critical hypo perfusion (Tmax > 6 s) (mean ± SD) (mL)	16.3 ± 4
Mean mismatch volume (mean ± SD) (mL)	13.5 ± 3.3

**Table 3:** Outcomes in patients treated before and after the implementation of AN.

Outcomes	AN− (*n* = 29)	AN+ (*n* = 47)	*p*-Value
Any Hemorrhagic transformation (%)	6.9%	10.6%	0.584
Symptomatic Intracranial Hemorrhage (%)	3.4%	0.0%	0.200
NIHSS-score 2 h, points (median, IQR)	2 (0.5–3.5)	3 (1–5.25)	0.230
NIHSS 24 h, points (median, IQR)	1 (0–4)	1.5 (0–4)	0.697
Discharge NIHSS (median, IQR)	0 (0–2.5)	0 (0–3)	0.977
3-month mRS-score, points (median, IQR)	2 (1–4)	3 (1–5)	0.614 ***
3-month Functional Independence (%) *	82.1%	89.1%	0.394
3-month Favorable Functional Outcome (%) **	75.0%	78.3%	0.746
3-month Mortality (%)	0.0%	4.3%	0.263

National Institute of Health Stroke Scale, NIHSS. * mRS-scores of 0–2. ** mRS-scores of 0–1. *** Cochran–Mantel–Haenszel test.

**Table 4:** Univariable and multivariable binary logistic regression analyses evaluating the association of the use of advanced neuroimaging in acute stroke chain pathway with outcomes.

Outcomes	Crude OR (95% CI)	*p*-Value	Adjusted * OR (95% CI)	*p*-Value
Any ICH	1.60 (0.29, 8.88)	0.586	1.30 (0.21, 8.01)	0.840
Functional Independence at 3 months	1.78 (0.47, 6.80)	0.398	12.89 (1.47, 113.00)	0.021
Favorable Functional Outcome at 3 months	1.20 (0.40, 3.63)	0.747	1.97 (0.54, 7.17)	0.304

Odds ratio, OR; confidence intervals, CI. * Adjusted for the age and baseline NIHSS score.

## Data Availability

The data presented in this study are available on request from the corresponding author. The data are not publicly available due to privacy.

## Supplementary Materials

The following are available online at https://www.mdpi.com/article/10.3390/jcm10132819/s1, Table S1: Outcomes in patients treated before and after the implementation of AN, confined in patients treated with IVT during the early time window.
