# Supplementary material for: Advanced Neuroimaging Preceding Intravenous Thrombolysis in Acute Ischemic Stroke Patients Is Safe and Effective
Source: J Clin Med. 2021 Jun 26;10(13):2819. doi: 10.3390/jcm10132819 (PMC8268827; doi:10.3390/jcm10132819)
Supplement: Supplementary file 1 [file jcm-10-02819-s001.zip › jcm-1263016-supplementary.pdf]

Journal of
Clinical Medicine

MDPI

Article

Supplementary Material: Advanced Neuroimaging Preceding Intravenous Thrombolysis in Acute Ischemic Stroke Patients Is Safe and Effective

Klearchos Psychogios, Apostolos Safouris, Odysseas Kargiotis, Georgios Magoufis, Athina Andrikopoulou, Ermioni Papageorgiou, Maria Chondrogianni, Georgios Papadimitropoulos, Eftihia Polyzogopoulou, Stavros Spiliopoulos, Elias Brountzos, Elefterios Stamboulis, Sotirios Giannopoulos and Georgios Tsivgoulis

Table S1. Outcomes in patients treated before and after the implementation of AN, confined in patients treated with IVT during the early time window.

	**AN− (*n* = 29)**	**AN+ (*n* = 36)**	***p*-Value**
Any Hemorrhagic Transformation (%)	6.9%	8.3%	0.829
Symptomatic Intracranial Hemorrhage (%)	3.4%	0.0%	0.262
NIHSS-score 2 hours, points (median, IQR)	2 (0.5–3.5)	2.5 (1–5)	0.346
NIHSS 24 hours, points (median, IQR)	1 (0–4)	2 (0–3)	0.740
Discharge NIHSS (median, IQR)	0 (0–2.5)	0 (0–2.75)	0.869
3-month Functional Independence (%) *	82.1%	94.4%	0.118
3-month Favorable Functional Outcome (%) **	75.0%	80.6%	0.594
3-month Mortality (%)	0%	0%	0.999

NIHSS: National Institute of Health Stroke Scale; * mRS-scores of 0–2; ** mRS-scores of 0–1.
